# Supplementary material for: Glucose control and outcomes in diabetic and nondiabetic patients treated with targeted temperature management after cardiac arrest
Source: PLoS One. 2024 Feb 8;19(2):e0298632. doi: 10.1371/journal.pone.0298632 (PMC10852315; doi:10.1371/journal.pone.0298632)
Supplement: S1 File — (PDF) [file pone.0298632.s001.pdf]

S1. Patient information and data used in analysis

No	Inclusion	Sex	Age	Age_65	previous_cardiac_arrest	AMI	angina_pectoris	CHF	HTN	DM	Renal disease	Arrest Etiology	Initial ryhthm	Witness	Bystander CPR	min	6m CPC	6m POOR	6m survival	Category	Initial HbA1c	Adm BST	At Least ONCE Hyperglycemia	At Least ONCE Hypoglycemia	Adm hyperglycemia	Time To Target Glucose	Glucose Reducing Rate	Insulin Dose To Target Glucose	Cumulative Insulin Requirement During 48h
	0: no<br>1: yes	0:F<br>1:M		0: <65<br>1: ≥65	0:no<br>1:yes	0:no<br>1:yes	0:no<br>1:yes	0:no<br>1:yes	0:no<br>1:yes	0:no<br>1:yes	0:no<br>1:yes	0: cardiac<br>1: non-cardiac	0: shockable<br>1:non-shockable	0:witnessed<br>1:unwtinessed	0:bystander CPR<br>1:no	Total anoxic time	1~5	0: good<br>1: poor	0:survive<br>1:no	1: No DM<br>2: poor controlled DM<br>3: well controlled DM<br>4: hidden DM			0: no<br>1: yes	0: no<br>1: yes	0: no<br>1: yes	hyper to normal<br>(hr)<br>hypo to normal			
1	0	1	62	0	0	0	0	0	0	0	0	1	1	0	1	32	4	1			.								
2	0	1	66	0	0	0	0	0	0	0	0	0	0	0	1	22	1	0			.								
3	0	0	52	0	0	0	0	0	0	0	0	1	1	0	1	27	5	1			.								
4	1	1	57	0	0	0	1	0	0	0	0	0	0	0	1	30	1	0	0	1	5.9	228	1	1	1	25	3.0	51	53
5	0	0	32	1	0	0	0	0	0	0	0	1	1	0	0	17	5	1			.								
6	1	1	34	0	0	0	0	0	0	0	0	1	1	0	1	54	4	1	0	1	5.6	103	1	1	0	0		0	67
7	1	1	47	0	0	0	0	0	0	0	0	0	1	0	1	20	5	1	1	1	5.6	274	1	0	1	1	198.0	0	0
8	1	1	67	0	0	0	0	0	0	0	0	1	1	1	1	37	5	1	1	1	5.4	360	1	0	1	6	33.7	0	0
9	0	1	48	0	0	0	0	0	0	0	0	0	1	0	0	49	5	1			.								
10	0	1	76	0	1	1	0	0	1	0	0	0	1	1	0	41	1	0			.								
11	0	1	39	1	0	0	0	0	1	1	0	1	1	1	1	12	.	.			5.3	102							
12	0	0	32	0	0	0	0	0	0	1	0	1	1	1	1	68	5	1			.								
13	1	1	54	1	0	1	0	0	1	1	0	0	1	0	1	12	4	1	0	2	6.1	449	1	1	1	15	19.5	28	28
14	0	1	53	0	0	0	0	0	0	0	0	0	0	0	1	23	1	0			.								
15	0	0	64	0	0	0	0	0	0	0	0	1	1	1	1	26	5	1			.								
16	1	1	73	0	0	0	0	0	0	0	0	0	1	0	1	25	1	0	0	1	5.1	230	1	1	1	1	137.0	0	36
17	1	1	59	0	0	0	0	0	1	0	0	0	0	1	1	36	2	0	0		6.2	364	1	0	1	5	40.0	32	53
18	0	0	74	0	0	0	0	0	0	0	0	1	1	1	1	35	5	1			.								
19	1	1	40	1	0	0	1	0	1	1	0	0	0	0	1	32	5	1	1	2	6.1	295	1	1	1	6	31.5	15	59
20	0	1	71	0	0	0	0	0	0	0	0	0	1	0	0	12	5	1			.								
21	0	1	48	1	0	0	0	0	1	1	1	1	1	0	1	33	5	1			.								
22	0	1	92	0	0	0	0	0	0	0	0	0	0	0	0	18	1	0			.								
23	0	1	20	1	0	0	0	0	1	0	0	0	1	0	1	30	5	1			.								
24	0	1	60	0	0	0	0	0	0	0	0	0	0	0	0	10	1	0			.								
25	0	1	62	1	0	0	0	0	1	0	0	1	1	1	1	29	5	1			.								
26	0	0	30	0	0	0	0	0	0	0	0	0	0	0	1	27	1	0			.								
27	0	1	64	0	0	0	0	0	0	0	0	0	0	1	1	15	1	0			.								
28	0	1	35	0	0	0	0	0	0	0	0	0	0	0	1	20	2	0			.								
29	0	1	69	0	0	0	0	0	0	0	0	0	0	0	1	15	1	0			.								
30	0	1	55	0	0	0	0	0	1	0	0	1	0	1	1	16	1	0			.								
31	0	1	49	0	0	0	0	0	0	0	0	0	0	0	1	93	5	1			.								
32	0	1	60	1	0	1	0	0	1	0	0	0	0	0	1	24	1	0			.								
33	0	1	41	0	0	0	0	0	0	0	0	0	0	1	0	.	1	0			.								
34	0	1	24	0	0	1	1	0	0	0	0	0	1	0	0	2	1	0			.								
35	1	0	37	0	0	0	0	0	0	0	0	0	1	0	0	12	5	1	1	1	5.4	155	1	0	0	0		0	16
36	0	0	65	0	0	0	0	0	0	0	0	0	0	0	1	23	1	0			.								
37	0	0	53	0	0	0	0	0	0	0	0	0	1	0	1	.	5	1			.								
38	0	1	56	0	0	0	0	0	0	1	0	0	1	1	1	.	5	1			.								
39	0	0	72	0	0	0	0	1	0	0	0	0	0	0	1	41	1	0			.								
40	1	0	31	0	0	0	0	0	1	0	0	0	1	0	0	23	5	1	1	1	5.3	314	1	1	1	4	52.5	12	33
41	0	1	48	0	0	0	1	0	1	0	0	0	0	0	0	22	1	0			.								
42	0	1	46	1	0	0	0	0	1	1	0	0	1	0	1	30	5	1			.								
43	0	1	75	0	0	0	0	0	0	0	0	0	0	0	0	11	1	0			.								
44	0	1	43	0	0	0	0	0	1	1	0	1	1	0	1	31	5	1			.								
45	0	1	54	0	0	0	0	0	1	1	1	0	1	0	1	10	5	1			.								
46	0	0	47	1	0	0	0	0	0	0	0	0	1	1	1	47	5	1			.								
47	0	1	70	0	0	0	0	0	0	0	0	1	1	1	1	25	4	1			.								
48	0	0	83	0	0	0	0	0	0	0	0	1	1	1	1	46	5	1			.								
49	1	1	59	0	0	0	0	0	0	0	0	0	1	1	1	18	1	0	0	1	5.6	233	1	0	1	6	13.8	0	0
50	0	1	32	1	0	0	0	0	0	0	0	0	1	1	1	25	5	1			.								
51	0	1	64	1	0	0	0	0	1	0	0	1	1	0	1	44	5	1			.								
52	0	1	52	0	0	0	0	0	0	0	0	1	1	0	0	28	5	1			.								
53	0	1	46	0	0	0	0	0	0	0	0	1	1	0	1	28	1	0			.								
54	0	0	32	0	0	0	0	0	0	0	0	1	1	0	0	8	1	0			.								
55	0	1	52	0	0	0	0	0	0	0	0	0	0	0	0	40	1	0			.								
56	0	1	43	0	0	0	0	0	1	0	0	0	1	0	0	61	4	1			.								
57	0	0	75	0	0	0	0	0	0	0	0	0	1	0	0	52	5	1			.								
58	0	1	46	0	0	0	0	0	0	1	0	0	1	0	0	24	5	1			.								
59	0	1	31	0	0	0	0	0	0	0	0	0	0	0	1	47	5	1			.								
60	0	1	57	1	0	0	0	0	0	0	0	0	1	1	1	33	5	1			.								
61	0	0	18	0	0	0	0	0	0	0	0	1	1	1	0	.	5	1			.								
62	0	1	73	0	0	0	0	1	0	0	0	0	0	1	1	20	1	0			.								
63	1	1	83	0	0	0	0	0	0	0	0	0	0	0	0	37	1	0	0	1	5.3	222	1	0	1	16	3.5	57	81
64	0	1	40	0	0	0	0	0	0	0	0	0	0	0	0	40	1	0			.								
65	1	0	46	1	0	0	0	0	1	1	0	0	1	0	0	10	5	1	1	3	5.2	263	1	0	1	13	8.3	21	41
66	1	0	45	1	0	0	0	0	0	1	0	1	1	1	1	40	1	0	0	2	6.4	179	1	0	0	0		0	35
67	1	1	21	0	0	0	0	1	0	0	0	0	0	0	0	59	3	1	0	1	5.1	161	1	0	0	0		0	87
68	0	1	46	0	0	0	0	0	0	0	0	1	1	1	1	53	5	1			.								
69	0	0	51	0	0	0	0	1	0	0	0	0	0	0	0	25	1	0			.								
70	0	1	54	0	0	0	0	0	0	0	0	0	0	0	0	12	1	0			.								
71	0	1	84	0	0	0	0	0	0	0	0	0	0	0	0	26	1	0			.								
72	1	1	40	0	0	1	0	0	0	0	0	0	1	0	0	32	1	0	0	1	4.9	112	1	0	0	0		0	0

73	0	0	28	0	0	0	0	0	0	0	0	1	1	0	0	25	5	1			.								
74	1	0	22	1	0	0	0	0	1	1	0	0	0	0	0	40	1	0	0	2	7.7	273	1	0	1	3	47.3	0	26
75	1	1	46	0	0	0	0	0	0	0	0	1	1	1	0	46	5	1	1	1	5.4	239	1	0	1	4	18.3	8	8
76	0	1	20	0	0	0	0	0	0	0	0	1	1	1	0	.	4	1			.								
77	1	0	49	0	0	0	0	0	0	0	0	0	0	0	1	17	1	0	0	1	4.5	234	1	0	1	7	9.0	12	12
78	1	1	39	0	0	0	0	1	0	0	0	0	0	0	1	43	5	1	1		6.8	250	1	0	1	9	8.0	20	28
79	1	1	69	0	0	0	0	0	0	0	0	0	0	0	1	65	1	0	0	1	5.1	186	1	0	1	1	38.0	0	104
80	1	1	58	0	0	0	0	0	0	0	0	0	0	0	0	9	1	0	0	1	5.9	232	1	0	1	3	31.3	0	0
81	1	1	69	0	0	0	0	0	0	0	0	0	0	0	0	15	1	0	0	1	5.4	264	1	0	1	4	24.0	0	0
82	1	1	66	1	0	0	0	0	0	0	0	0	0	0	0	12	1	0	0	1	5.7	265	1	0	1	3	38.3	0	0
83	1	1	72	0	1	0	0	1	1	1	0	0	1	1	0	24	5	1	1	2	12.1	656	1	0	1	.	.	0	50
84	1	1	35	1	0	0	0	0	0	0	0	1	1	0	0	63	5	1	1		7	301	1	0	1	8	20.3	0	16
85	1	1	43	1	0	0	0	0	0	1	1	0	1	0	1	25	5	1	1	2	6.5	177	1	1	0	0		0	0
86	0	0	74	1	0	0	0	0	0	0	0	0	0	0	0	5	1	0			.								
87	1	1	33	0	0	0	0	0	0	0	0	1	1	1	1	51	5	1	1	1	5.4	359	1	1	1	9	20.4	24	26
88	1	0	44	0	0	0	0	0	0	0	0	1	1	1	1	34	5	1	1	1	4.8	302	1	0	1	7	22.9	24	24
89	1	1	68	1	0	0	0	0	1	1	0	0	1	0	0	26	5	1	1	2	6.7	471	1	0	1	14	22.0	51	55
90	1	1	51	0	0	0	0	0	0	0	0	1	1	1	1	31	4	1	0	1	4.6	249	1	0	1	11	9.3	0	4
91	1	0	70	0	0	0	0	0	0	0	0	1	1	1	1	59	5	1	1	1	5.1	186	1	0	1	7	0.9	0	8
92	1	0	69	1	0	0	0	0	0	1	0	0	1	0	0	10	1	0	0	2	9	647	1	0	1	44	10.7	297	305
93	1	1	61	0	0	0	0	0	0	0	0	0	0	0	0	30	1	0	0	1	5.5	323	1	0	1	3	49.0	0	0
94	1	1	47	1	0	1	0	0	1	1	1	0	1	0	0	23	5	1	1	3	5	457	1	1	1	3	92.3	4	6
95	1	0	70	1	0	0	0	0	1	1	0	1	1	0	1	18	5	1	1	2	6.8	241	1	0	1	12	7.1	11	21
96	1	1	59	0	0	0	0	0	1	1	0	0	1	1	1	62	5	1	1	2	6.9	259	1	0	1	14	6.1	8	8
97	1	1	82	0	0	0	0	0	0	0	0	0	1	0	0	25	5	1	1	1	5.8	56	0	1	0	20		67	73
98	1	1	30	1	0	0	0	0	0	0	0	0	1	0	0	35	5	1	1	1	5.6	332	1	0	1	8	29.6	15	15
99	1	1	75	0	0	0	0	0	1	0	0	0	0	0	1	18	1	0	0		6.6	242	1	0	1	8	8.4	12	33
100	1	0	47	1	0	0	0	0	0	0	0	1	1	0	1	34	5	1	1	1	5.4	262	1	0	1	7	11.7	7	60.5
101	1	0	70	0	0	0	0	0	0	0	0	0	0	0	0	27	2	0	0	1	5.3	282	1	0	1	10	10.2	28	106
102	1	1	41	1	0	0	0	0	1	0	1	1	1	0	1	49	5	1	1	1	4.6	153	0	1	0	0		0	0
103	1	0	45	0	0	0	0	0	0	0	0	1	1	1	1	36	4	1	0	1	5.1	291	1	1	1	4	35.5	6.5	30
104	1	1	46	1	0	0	1	0	1	0	0	0	0	0	1	7	1	0	0		6.2	188	1	0	1	11	5.7	8	8
105	1	0	58	0	0	0	0	0	0	0	0	1	1	0	0	25	5	1	1	1	5.9	472	1	0	1	4	73.3	0	0
106	1	1	73	0	0	0	0	0	0	0	0	0	0	0	0	22	1	0	0	1	5.4	249	1	0	1	3	23.7	0	0
107	1	0	73	0	0	0	0	0	0	0	0	0	1	0	0	35	4	1	0	1	4.9	348	1	0	1	5	40.0	0	0
108	1	0	38	0	0	0	0	0	0	0	0	0	0	0	1	44	5	1	1	1	5.3	459	1	0	1	7	41.9	25	59.5
109	1	1	58	1	0	1	0	0	0	0	0	0	1	0	0	30	5	1	1		6.4	114	1	1	0	0		0	547
110	1	1	63	1	0	0	0	1	1	1	0	0	1	0	1	39	5	1	1	2	6	222	1	0	1	8	10.6	6	6
111	1	0	52	0	0	0	0	0	0	0	0	0	0	0	0	25	5	1	1	1	5.4	278	1	1	1	6	19.5	13	27
112	1	0	73	0	0	0	0	0	1	0	0	0	0	1	1	61	5	1	1	1	5.7	290	1	0	1	24	5.2	51	67
113	1	0	47	0	0	0	0	0	0	0	0	1	1	0	1	25	5	1	1		6	223	1	1	1	5	20.0	7	33.5
114	1	1	42	0	0	0	0	0	0	0	0	0	0	0	0	27	3	1	0	1	4.6	184	1	0	1	7	1.1	6	17
115	1	0	72	1	0	0	0	0	1	1	1	0	1	1	1	37	5	1	1	3	5.6	286	1	0	1	11	13.5	99	107
116	1	1	56	0	0	0	0	0	0	0	0	0	0	0	1	27	1	0	0	1	5.5	337	1	0	1	4	52.3	8	8
117	1	1	56	0	0	0	0	0	0	0	0	0	0	0	0	11	1	0	0		7.1	195	1	0	1	6	5.5	4	8
118	1	0	48	1	0	0	0	0	0	0	0	0	1	1	0	100	5	1	1	1	5.7	288	1	1	1	4	37.8	5	8
119	1	1	38	0	0	0	0	0	0	0	0	0	1	0	0	3	1	0	0	1	5.5	288	1	0	1	4	45.3	8	8
120	1	1	51	0	0	0	0	0	0	1	0	1	1	0	0	5	5	1	1	3	5.5	310	1	0	1	5	41.0	15	33
121	1	1	38	0	0	0	0	0	1	0	0	0	0	0	0	31	3	1	0		6.3	341	1	0	1	7	24.1	19	28
122	1	1	60	0	0	0	0	0	0	0	0	0	0	0	0	10	1	0	0		6	158	1	0	0	0		0	95
123	1	1	62	0	0	0	0	0	0	0	0	0	1	0	0	24	5	1	1	1	5.2	220	1	1	1	6	11.3	6	18
124	1	0	56	0	0	0	0	0	0	0	0	1	1	1	1	41	5	1	1	1	5.4	206	1	0	1	2	22.5	3	18
125	1	1	78	0	0	0	0	0	0	0	0	0	1	1	1	22	5	1	1	1	5.5	214	1	0	1	2	22.5	0	0
126	1	1	54	0	0	1	0	0	1	1	0	1	1	1	1	42	5	1	1	3	5.8	436	1	1	1	6	45.2	12	14
127	1	1	78	0	0	0	0	0	1	0	0	1	1	1	0	39	5	1	1	1	5.7	322	1	0	1	28	5.8	57	61
128	1	1	45	1	0	0	0	0	1	0	0	0	1	0	0	51	5	1	1		6.5	181	1	0	1	9	1.8	21	41
129	1	1	66	0	0	1	0	0	0	0	0	0	0	1	1	>27	5	1	1	1	5.6	122	1	0	0	0		0	0
130	1	1	20	1	0	0	0	0	1	0	0	0	1	1	1	70	5	1	1	1	5.7	87	0	1	0	0		0	0
131	1	1	47	0	0	0	0	0	1	0	0	0	0	0	0	64	5	1	1	1	5.5	342	1	0	1	9	18.7	18	54
132	1	1	70	1	0	1	0	0	1	1	0	0	1	0	0	27	3	1	0	2	6.1	193	1	0	1	18	1.3	30	44
133	1	0	70	0	0	0	0	0	0	0	0	1	1	1	1	41	5	1	1	1	5.5	299	1	0	1	44	2.9	156	164
134	0	1	75	0	0	0	0	0	0	0	0	0	0	0	0	59	1	0			.								
135	1	1	73	1	0	0	1	0	0	1	1	0	1	0	0	50	5	1	1	3	5.6	319	1	1	1	13	13.7	34	57
136	1	1	43	1	0	0	0	0	1	1	0	0	1	1	1	18	5	1	1	2	11.6	424	1	0	1	14	19.1	51	67
137	1	1	68	1	0	0	1	0	1	1	1	0	1	0	0	49	5	1	1	2	10	339	1	1	1	7	26.4	40	46
138	1	1	18	1	0	0	0	0	0	0	0	1	1	0	1	55	5	1	1	1	5.6	428	1	1	1	5	62.6	0	0
139	0	0	57	0	0	0	0	0	0	1	0	0	1	1	1	23	5	1			.								
140	1	0	64	1	0	0	0	0	0	0	0	0	1	1	0	25	5	1	1	1	5.6	350	1	0	1	8	24.5	8	25.5
141	1	1	78	0	0	0	0	0	0	0	0	0	0	0	0	15	1	0	0	1	5.4	193	1	0	1	6	6.7	11	25
142	1	1	42	0	0	0	0	0	1	1	0	0	0	1	0	35	5	1	1	2	11.3	515	1	0	1	12	29.0	24	70
143	1	1	53	0	0	0	0	0	0	1	0	0	0	0	1	47	5	1	1	2	7.6	302	1	0	1	4	34.5	8	12
144	1	0	62	1	0	0	0	0	1	0	0	0	1	0	1	54	5	1	1	1	5.7	448	1	1	1	10	27.2	45	51
145	0	0	61	0	0	0	0	0	1	0	0	0	1	0	0	6	4	1			.								
146	1	1	69	0	0	0	0	0	1	0	0	0	0	0	0	45	5	1	1	1	5.5	136	1	0	0	0		0	2
147	1	1	83	0	0	0	0	0	0	1	0	0	1	0	0	24	5	1	1	2	6.9	273	1	0	1	7	16.4	6	38
148	0	1	44	0	0	0	0	0	0	0	0	0	0	0	1	12	1	0			.								
149	1	1	50	1	0	1	1	0	1	1	0	0	1	0	1	37	5	1	1	2	6.4	257	1	0	1	6	13.0	8	44
150	1	1	58	1	0	0	0	0	1	0	0	0	1	1	1	50	5	1	1		6.4	206	1	0	1	7	11.4	0	0
151	1	1	45	0	0	0	0	0	0	0	0	1	1	0	1	59	5	1	1	1	4.8	421	1	0	1	5	54.0	0	0
152	1	1	33	0	0	0	0	0	0	0	0	0	0	0	0	38	1	0	0		6.1	376	1	0	1	10	19.7	33	57.5
153	1	0	30	0	0	0	0	0	0	0	0	0	1	0	1	28	1	0	0		6.3	387	1	0	1	14	15.1	14	36

154	1	1	64	0	0	0	0	0	0	0	0	0	0	0	0	20	1	0	0	1	4.7	267	1	0	1	7	13.3	3	18
155	1	0	66	0	0	0	0	0	0	0	0	1	1	0	0	55	5	1	1	1	5.4	357	1	0	1	7	25.9	22	28.5
156	1	0	56	0	0	0	0	0	0	0	0	1	1	1	1	14	3	1	0	1	5.1	288	1	0	1	7	20.6	0	0
157	0	1	61	0	0	0	0	0	1	0	0	0	0	0	0	35	5	1			.								
158	1	1	57	1	0	1	0	0	1	1	0	0	0	0	0	31	5	1	1	2	7.1	259	1	0	1	.	.	0	0
159	1	1	71	0	0	0	0	0	0	0	0	1	1	1	1	41	5	1	1	1	5.1	169	1	0	0	0		0	0
160	1	1	47	0	0	1	0	0	1	1	0	0	1	0	1	42	5	1	1	2	6.4	304	1	0	1	10	15.5	33	86
161	1	1	47	0	0	0	0	0	1	0	0	0	1	0	0	36	4	1	0	1	5.3	311	1	0	1	9	17.8	15	74
162	1	1	44	1	0	0	0	0	1	0	0	0	1	1	1	38	5	1	1		7.3	9	0	1	1	4		0	0
163	1	1	49	0	0	0	1	0	1	0	0	0	1	0	0	28	5	1	1	1	5.8	280	1	1	1	4	41.8	29	58
164	1	1	23	0	0	0	0	0	0	0	0	0	0	0	0	68	5	1	1	1	5.2	452	1	1	1	12	23.2	62	83
165	1	1	74	0	0	0	0	0	0	0	0	0	0	0	0	37	1	0	0		6.3	371	1	0	1	1	201.0	6	32
166	1	0	25	0	0	0	0	0	0	0	0	1	0	0	1	26	5	1	1	1	5.1	301	1	0	1	9	16.4	0	65
167	1	1	53	0	0	0	0	0	0	0	0	0	0	1	1	38	1	0	0	1	5.7	242	1	0	1	3	25.7	0	0
168	1	0	67	1	0	0	0	0	0	0	0	1	1	1	0	41	5	1	1	1	5.7	344	1	0	1	8	22.0	9	25
169	1	0	53	0	0	0	0	0	0	0	0	0	1	1	0	42	5	1	1		6.2	222	1	0	1	8	11.9	0	0
170	0	1	61	0	0	0	0	0	1	0	0	0	0	0	0	10	1	0			.								
171	1	1	90	1	0	0	0	0	0	0	0	0	0	0	0	10	1	0	0	1	5.2	125	1	0	0	0		0	0
172	1	1	34	0	0	1	0	0	0	0	0	0	0	0	1	9	1	0	0		6.4	178	1	0	0	0		0	55
173	1	1	72	0	0	1	0	0	1	0	0	0	0	0	0	19	5	1	1		6.1	273	1	1	1	11	8.5	24	102
174	1	0	54	1	0	0	0	0	1	0	1	0	0	0	0	30	5	1	1	1	5.6	301	1	0	1	5	39.0	3	3
175	1	1	54	0	0	0	0	0	0	0	0	0	0	0	0	11	1	0	0	1	5.3	255	1	0	1	19	4.3	28	38
176	1	1	63	1	0	0	0	0	1	0	0	0	0	0	0	53	5	1	1		7	339	1	0	1	.	.	0	0
177	1	1	66	0	0	0	0	0	0	0	0	0	0	0	0	41	5	1	1	1	5.7	394	1	0	1	6	36.8	7	53
178	1	1	73	0	0	0	0	0	0	0	0	1	1	1	0	26	4	1	0	1	5.6	376	1	0	1	4	61.5	6	20
179	1	1	62	0	0	0	0	0	1	0	0	0	0	0	0	20	1	0	0	1	5.3	277	1	1	1	4	24.3	8	50.5
180	1	1	73	1	0	0	0	0	1	1	0	0	0	0	0	40	5	1	1	2	7	292	1	0	1	6	20.3	14	32
181	1	0	56	1	0	0	0	0	0	0	0	0	1	0	0	33	4	1	0	1	5	379	1	0	1	8	27.0	15	67
182	0	0	54	0	0	1	0	0	0	1	0	0	1	0	1	31	5	1			.								
183	1	1	32	1	0	0	1	0	0	1	1	0	1	0	0	6	5	1	1	2	6.9	204	1	0	1	2	22.5	0	55
184	1	1	78	0	0	0	0	0	0	0	0	0	1	0	0	62	5	1	1	1	5.8	299	1	0	1	2	67.0	0	0
185	1	1	69	0	0	0	0	1	0	0	0	0	1	0	0	33	5	1	1	1	5.3	121	1	0	0	0		0	5
186	0	1	68	0	1	0	0	0	0	0	0	0	0	0	1	.	4	1			.								
187	1	1	80	1	0	0	0	0	0	0	0	1	1	0	0	35	5	1	1	1	4.7	167	1	1	0	0		0	0
188	1	0	43	1	0	0	0	0	0	0	0	1	1	1	0	30	5	1	1	1	5.7	433	1	0	1	6	44.3	63	85
189	1	1	47	1	0	0	0	0	0	0	0	1	1	0	0	18	5	1	1		6.2	191	1	0	1	4	4.3	4	10
190	1	1	57	1	0	0	0	0	1	0	0	0	1	0	1	13	5	1	1	1	5.3	306	1	0	1	6	21.3	9	13
191	1	1	57	0	0	0	0	0	1	0	0	0	0	0	0	20	1	0	0	1	5.5	311	1	0	1	9	14.8	26	36
192	1	1	34	0	0	1	0	0	1	0	0	0	0	0	0	40	1	0	0		6.5	422	1	0	1	17	14.5	58	110
193	1	1	57	0	0	0	0	0	1	0	0	0	0	0	1	16	1	0	0	1	5.3	216	1	1	1	6	9.3	3	11
194	1	0	38	0	0	0	0	0	0	0	0	0	0	0	0	42	1	0	0		6	264	1	0	1	7	15.4	14	32
195	1	1	56	0	0	0	0	0	0	0	0	0	1	0	0	61	1	0	0	1	5.3	141	1	0	0	0		0	0
196	1	1	48	0	0	0	0	0	0	0	0	1	1	0	0	12	5	1	1	1	5.3	163	1	0	0	0		0	0
197	1	1	51	0	0	0	0	0	1	0	0	0	0	0	1	27	1	0	0		6.1	159	0	0	0	0		0	0
198	1	0	72	0	0	0	0	0	0	0	0	0	0	0	0	31	5	1	1	1	5.9	234	1	0	1	2	41.0	0	0
199	1	0	32	0	0	0	1	0	0	0	0	0	0	0	0	57	1	0	0	1	5.8	186	1	0	1	3	3.3	0	0
200	1	1	39	0	0	0	0	0	0	0	0	0	0	0	0	64	3	1	0	1	5.2	294	1	0	1	4	34.0	8	50
201	1	0	72	1	0	0	0	0	0	1	0	1	1	0	1	35	5	1	1	3	5.7	134	0	0	0	0		0	20
202	1	0	89	0	0	0	0	0	0	0	0	1	1	1	1	52	5	1	1	1	5.2	251	1	0	1	8	8.9	0	0
203	1	0	32	0	0	0	0	0	0	0	0	0	0	1	0	33	1	0	0	1	4.6	275	1	0	1	5	24.0	0	47
204	0	1	54	1	0	1	0	0	0	1	0	0	0	0	1	48	5	1			.								
205	1	0	73	1	0	0	0	0	1	1	0	1	1	0	0	52	5	1	1	2	6.3	108	1	0	0	0		0	49
206	1	1	23	0	0	0	0	0	0	0	0	1	1	1	0	40	5	1	1	1	5.6	404	1	0	1	3	76.0	0	4
207	1	1	73	0	0	0	0	0	0	0	0	0	0	0	1	2	1	0	0	1	5.8	258	1	0	1	10	9.7	0	0
208	1	1	26	1	0	0	0	0	0	0	0	1	1	0	0	37	5	1	1	1	5.7	182	1	1	1	2	17.5	0	0
209	1	0	67	0	0	0	0	0	0	0	0	0	0	0	0	21	1	0	0	1	4.6	256	1	0	1	5	25.8	3	5
210	1	1	44	1	0	1	0	0	1	0	0	0	0	1	1	35	5	1	1	1	5.5	425	1	0	1	8	35.9	1	1
211	1	0	67	0	0	0	0	0	0	0	0	0	0	1	0	11	1	0	0	1	5.5	218	1	0	1	1	78.0	0	0
212	1	1	51	1	0	0	0	0	1	0	0	0	0	0	0	11	1	0	0	1	5.4	205	1	0	1	2	20.0	0	0
213	1	0	48	0	0	0	0	0	0	0	0	1	1	1	1	39	5	1	1		6	584	1	0	1	25	16.9	271	291.5
214	1	1	52	1	0	0	1	0	0	0	0	0	1	0	0	8	1	0	0		6.8	192	1	0	1	6	10.5	5	19
215	1	1	74	0	0	0	0	0	0	0	0	0	0	0	0	40	3	1	0	1	5.7	400	1	0	1	4	57.0	12	50
216	1	1	86	0	0	0	0	0	0	1	0	0	0	0	1	30	1	0	0	2	6.8	344	1	0	1	9	18.3	42	126.5
217	1	1	46	0	0	0	0	0	1	1	0	1	1	1	1	64	5	1	1	2	6.6	283	1	0	1	5	35.8	0	10
218	1	1	58	1	0	0	0	0	1	1	0	1	1	1	1	8	5	1	1	3	5.9	302	1	0	1	12	11.3	21	27
219	1	1	71	1	0	0	0	0	1	1	1	1	1	0	1	12	5	1	1	2	7.7	413	1	0	1	15	18.2	16	30
220	1	1	42	0	0	0	0	0	1	1	0	1	1	0	0	62	5	1	1	3	5.9	180	1	0	0	0		0	10
221	1	1	72	0	0	0	0	0	0	1	1	1	1	1	1	42	5	1	1	2	6.3	314	1	1	1	8	16.9	13	70
222	1	0	55	1	0	0	0	0	1	0	0	0	0	0	1	36	5	1	1	1	5.7	309	1	0	1	5	28.2	10	26
223	1	1	70	0	0	0	0	0	1	0	0	1	0	0	0	15	5	1	1	1	4.9	252	1	0	1	1	97.0	0	0
224	1	1	71	1	0	0	1	0	1	1	0	0	0	1	0	30	5	1	1	2	9	364	1	0	1	9	23.8	25	39
225	0	1	27	0	0	0	0	0	0	0	0	0	0	0	0	58	.	.			10	568							
226	1	1	39	1	0	0	0	0	0	1	0	0	1	0	0	33	5	1	1	2	7.1	278	1	0	1	6	18.8	6	28
227	1	1	32	1	0	0	0	0	0	0	0	0	0	0	0	9	1	0	0		6.5	185	1	0	1	3	2.0	0	74
228	1	0	13	0	0	0	0	0	0	0	0	1	1	1	0	37	5	1	1	1	5.3	200	1	0	1	5	18.0	0	0
229	1	0	58	0	0	0	1	0	0	0	0	0	1	0	1	9	1	0	0		6	270	1	0	1	10	14.0	0	0
230	1	1	63	0	0	0	0	0	1	0	0	0	0	0	0	19	1	0	0	1	5.2	251	1	0	1	13	5.7	30	50
231	1	1	46	0	0	0	0	0	0	0	0	1	1	0	0	74	5	1	1	1	5.5	160	0	1	0	0		0	0
232	0	1	81	0	0	0	0	0	0	0	0	1	1	0	0	20	5	1			.	367							
233	1	1	55	0	0	1	0	0	0	0	0	0	1	0	1	15	1	0	0	1	5.4	207	1	0	1	9	3.0	0	2
234	0	1	41	0	0	0	0	0	0	0	0	1	1	0	0	45	4	1			.	228							

235	0	1	57	1	0	0	0	0	1	1	0	0	0	0	0	41	5	1			.	346							
236	1	1	57	0	0	0	0	0	1	1	0	0	1	0	0	10	1	0	0	2	8.7	335	1	0	1	19	8.7	71	89
237	1	1	73	0	0	0	0	0	0	0	0	1	1	1	1	59	5	1	1		12.7	308	1	0	1	13	12.0	31	37
238	1	1	43	0	0	0	0	0	1	0	0	0	0	0	0	72	5	1	1	1	5.7	449	1	0	1	4	70.5	8	76
239	1	1	75	0	0	0	0	0	1	1	1	1	1	1	0	138	5	1	1	3	5.3	159	1	0	0	0		0	0
240	1	1	44	1	0	0	0	0	0	0	0	1	1	1	1	49	5	1	1	1	5.2	140	1	0	0	0		0	16
241	1	1	86	0	0	0	0	0	0	0	0	0	0	0	1	42	1	0	0	1	5.5	366	1	0	1	19	10.1	51	67
242	1	1	50	1	0	0	0	0	0	1	0	1	1	0	1	47	5	1	1	2	6.9	311	1	0	1	3	44.0	12	62
243	1	0	76	0	0	0	0	0	0	0	0	0	0	0	0	33	1	0	0	1	5.3	365	1	0	1	11	17.2	8	8
244	1	1	43	1	0	0	0	0	0	0	0	1	1	0	1	39	5	1	1	1	5.2	179	0	0	0	0		0	0
245	1	0	49	0	0	0	0	0	0	0	0	1	1	1	0	30	5	1	1	1	5.8	387	1	0	1	6	36.5	10	10
246	1	0	49	1	0	0	0	0	1	0	0	1	1	0	1	38	5	1	1	1	5.8	285	1	0	1	6	21.7	14	36
247	1	1	44	0	0	0	0	0	0	0	0	1	1	1	0	87	5	1	1	1	5.1	254	1	1	1	2	39.5	2	4
248	1	0	83	0	0	0	0	0	0	0	0	1	1	0	0	9	5	1	1	1	5.4	369	1	0	1	6	37.3	4	4
249	1	1	77	0	0	0	0	0	0	0	0	0	1	0	0	55	5	1	1	1	5.4	147	1	0	0	0		0	32
250	1	1	86	0	0	0	0	1	0	0	0	0	1	0	0	11	5	1	1	1	5.8	32	0	1	0	7		0	2
251	1	1	38	1	0	0	0	0	0	0	1	0	1	0	0	71	5	1	1	1	5.8	115	0	0	0	0		0	0
252	1	1	72	1	0	0	0	0	0	1	1	1	1	0	0	15	5	1	1	2	7.5	181	1	0	1	8	4.3	13	29
253	1	0	46	1	0	0	0	0	0	0	0	0	1	0	0	34	5	1	1	1	4.9	27	0	1	0	6		0	0
254	1	1	53	0	0	0	0	0	0	0	0	1	1	1	0	53	5	1	1	1	5.1	288	1	0	1	7	16.0	12	59
255	1	1	30	1	0	0	0	0	1	0	0	1	1	1	1	48	5	1	1	1	5.3	310	1	0	1	13	11.2	30	74
256	1	0	24	0	0	0	0	0	0	0	0	1	1	1	0	41	5	1	1	1	4.9	195	1	0	1	12	2.8	20	60
257	1	1	65	0	0	1	0	0	0	1	1	0	1	0	0	38	5	1	1	2	8.3	174	1	0	0	0		0	120
258	1	0	26	0	0	0	0	0	0	0	0	1	1	1	0	45	5	1	1	1	5.4	246	1	0	1	10	13.8	0	33
259	1	1	68	0	0	0	0	0	0	0	0	1	1	0	1	52	5	1	1	1	5.2	688	1	1	1	1	604.0	0	0
260	1	0	75	1	0	0	0	0	1	1	0	0	1	0	1	22	5	1	1	2	7.8	550	1	0	1	30	12.5	104	138
261	1	0	66	0	0	0	0	0	0	0	0	0	0	0	0	8	1	0	0	1	5.1	180	1	0	0	0		0	0
262	1	0	70	1	0	0	0	0	1	0	0	0	1	1	0	29	5	1	1	1	5.3	418	1	0	1	14	18.2	25	55
263	1	1	47	1	0	0	0	0	1	1	0	0	1	0	0	4	5	1	1	2	6.5	269	1	0	1	6	21.0	6	30
264	1	1	34	1	0	0	0	0	0	0	0	0	0	0	0	75	1	0	0	4	6.1	306	1	0	1	8	18.9	19	53
265	1	1	44	1	0	0	0	0	1	0	0	1	1	1	0	309	5	1	1	1	5.4	288	1	0	1	8	17.9	0	10
266	1	1	45	0	0	0	0	0	0	0	0	1	1	1	0	77	5	1	1	1	5.8	325	1	0	1	5	43.6	6	24
267	1	1	46	0	0	0	0	0	0	0	0	0	1	0	1	14	1	0	0	1	5.4	276	1	0	1	4	33.0	0	0
268	1	0	48	0	0	0	0	0	0	0	0	1	1	1	1	30	1	0	0	1	4.8	214	1	0	1	2	32.5	0	0
269	1	1	49	0	0	0	0	0	0	0	0	1	1	0	1	22	4	1	0	1	4.4	326	1	0	1	1	157.0	0	0
270	1	1	21	0	0	0	0	0	0	0	0	1	1	1	1	41	5	1	1	1	4.9	244	1	0	1	9	7.7	0	0
271	1	0	73	0	0	0	0	0	0	0	0	1	1	1	0	4	3	1	0	1	5.6	210	1	0	1	3	13.0	0	8
272	1	0	69	0	0	0	0	0	0	0	0	0	1	1	1	14	1	0	0	1	5.3	180	1	0	0	0		0	0
273	1	1	33	0	0	0	0	0	0	0	0	1	0	1	0	41	5	1	1	1	5.8	285	1	1	1	6	21.0	0	51
274	1	1	45	1	0	0	0	0	1	1	0	1	1	0	0	30	5	1	1	2	6.7	161	1	1	0	0		0	470
275	1	1	81	1	0	0	0	0	1	1	1	1	1	0	0	10	1	0	0	2	7.7	257	1	0	1	7	14.0	13	68.5
276	0	0	46	0	0	0	0	0	0	0	0	0	1	0	0	16	1	0			.	478							
277	1	1	49	0	0	0	0	0	0	0	0	1	1	1	0	36	5	1	1	1	5.4	338	1	0	1	7	24.4	3	3
278	1	0	63	1	0	0	0	0	1	0	0	0	1	1	1	40	5	1	1	4	6.5	181	1	1	1	3	13.3	0	0
279	1	1	33	0	0	0	0	0	0	0	0	1	1	1	0	25	1	0	0	1	5.1	233	1	0	1	6	21.5	0	0
280	1	1	20	0	0	0	0	0	1	1	1	1	1	0	1	46	5	1	1	2	6.2	353	1	0	1	6	31.8	41	43
281	1	1	58	0	0	0	0	0	0	0	0	0	1	1	0	53	5	1	1	1	4.5	446	1	0	1	2	182.0	0	0
282	1	0	78	0	0	0	0	0	0	0	0	1	0	1	1	46	5	1	1	1	5.3	284	1	0	1	6	20.0	3	3
283	1	1	66	0	0	0	0	0	0	0	0	1	1	1	0	49	5	1	1	1	5.5	200	1	0	1	3	16.7	3	3
284	1	1	81	0	0	0	0	0	0	0	0	0	1	0	1	42	5	1	1	1	5.8	355	1	0	1	5	37.2	7	9
285	1	1	65	1	0	0	1	0	1	1	0	0	0	0	1	31	1	0	0	2	7.8	291	1	0	1	6	19.2	15	29
286	1	1	58	1	1	1	0	0	1	1	0	0	1	0	0	32	3	1	0	2	7.2	375	1	0	1	5	40.4	14	71
287	1	0	81	1	0	0	0	0	0	1	0	1	1	0	0	10	5	1	1	2	7.1	365	1	1	1	17	11.2	34	63
288	1	0	38	1	0	0	0	0	1	0	0	0	0	0	0	30	2	0	0	1	5.6	385	1	0	1	8	28.6	34	44
289	1	1	58	0	0	0	0	0	1	1	0	0	1	0	0	13	1	0	0	2	6.9	201	1	0	1	7	3.9	12	50
290	1	1	71	1	0	0	0	0	0	0	0	1	1	1	1	65	5	1	1	4	6	408	1	1	1	6	45.3	0	0
291	1	0	44	0	0	0	0	0	0	0	0	1	1	1	1	32	5	1	1	1	5	255	1	0	1	5	29.6	6	36
292	1	0	65	0	0	0	0	0	1	0	0	1	1	0	0	42	5	1	1	4	6.4	362	1	0	1	16	11.5	61	91
293	1	1	72	1	0	0	0	0	0	1	1	0	1	1	1	52	5	1	1	3	5.7	116	1	0	0	0		0	0
294	1	1	62	0	0	0	0	0	0	0	0	0	0	0	0	77	5	1	1	1	5.3	398	1	0	1	9	24.7	20	20
295	1	0	61	1	0	0	0	0	0	1	0	0	0	0	0	32	5	1	1	3	5.4	493	1	0	1	.	.	0	42
296	1	1	80	1	0	0	0	0	0	0	0	1	1	0	1	52	5	1	1	1	5.6	99	1	0	0	0		0	0
297	1	0	58	0	0	0	0	0	0	1	0	0	1	0	0	16	4	1	0	2	6.8	347	1	0	1	23	8.6	65	114
298	1	1	67	0	0	1	0	0	0	0	0	0	0	0	0	32	1	0	0	1	5.6	96	0	0	0	0		0	0
299	1	1	47	1	0	0	0	0	1	0	0	0	0	0	0	10	5	1	1	1	5.1	144	1	0	0	0		0	34
300	1	1	82	0	0	0	0	0	0	0	0	1	1	0	0	33	5	1	1	1	5.1	116	1	0	0	0		0	0
301	1	1	32	1	0	0	0	0	1	0	0	0	0	0	0	14	1	0	0	1	5.6	185	1	0	1	6	3.3	0	8
302	1	1	67	0	0	0	0	1	0	0	0	0	1	0	0	46	1	0	0	1	5.6	260	1	0	1	4	20.3	16	60
303	1	1	53	1	0	0	0	0	0	0	0	1	1	0	0	26	5	1	1	1	5.4	235	1	0	1	1	56.0	0	8
304	1	1	74	0	0	0	0	0	0	0	0	1	1	1	0	56	5	1	1	1	5.5	356	1	0	1	3	64.0	9	11
305	1	1	16	1	0	1	0	0	1	1	0	0	0	0	1	40	5	1	1	3	5.9	150	1	0	0	0		0	34
306	1	1	52	0	0	0	0	0	0	0	0	1	1	1	0	.	5	1	1	4	6.1	320	1	1	1	1	210.0	0	74
307	1	0	84	1	0	0	0	0	1	0	0	1	1	1	0	25	5	1	1	1	5.5	318	1	0	1	10	14.9	9	11
308	1	0	76	0	0	0	0	0	0	0	0	0	0	0	0	10	1	0	0	1	5.2	173	1	0	0	0		0	0
309	1	0	78	0	0	0	0	0	0	1	0	1	1	0	0	18	3	1	0	3	5.7	231	1	0	1	7	7.9	0	34
310	0	1	67	1	0	0	0	1	1	1	1	0	1	0	0	9	5	1			.	179							
311	1	1	54	1	0	0	0	0	1	0	0	1	1	0	1	35	5	1	1	4	6.2	196	1	1	1	1	68.0	0	0
312	1	1	42	1	0	0	1	0	1	1	0	1	1	0	0	11	5	1	1	2	7.1	522	1	1	1	10	34.9	35	56
313	0	1	66	1	0	0	0	0	0	0	0	1	1	1	1	33	5	1			.	366							
314	1	1	18	0	0	0	0	0	0	0	0	0	1	0	1	54	5	1	1	4	15.2	298	1	0	1	22	6.9	57	65
315	1	1	46	0	0	0	0	0	1	1	0	1	0	0	1	112	5	1	1	2	7.5	518	1	0	1	3	122.7	0	134

316	1	1	82	1	0	0	0	0	0	0	0	0	0	0	1	9	1	0	0	1	5.1	171	0	0	0	0		0	5
317	1	1	84	0	0	0	0	0	0	0	0	0	0	0	0	50	2	0	0	1	5.3	245	1	0	1	28	2.6	114	136
318	1	1	69	0	0	0	0	0	0	0	0	1	1	1	0	51	5	1	1	1	5.6	398	1	0	1	7	32.0	11	33
319	1	1	64	1	0	0	0	0	0	1	0	0	1	0	1	36	5	1	1	2	6.9	243	1	1	1	11	9.3	21	23
320	1	1	46	1	0	0	0	0	0	0	0	1	1	1	0	43	5	1	1	1	5.7	319	1	0	1	2	83.0	8	14
321	1	1	49	1	0	0	0	0	0	1	0	1	1	1	1	68	5	1	1	2	6.9	340	1	0	1	18	13.5	64	64
322	1	1	69	0	0	0	0	0	1	0	0	0	0	0	0	38	5	1	1	4	6.5	181	1	0	1	.	.	0	22
323	1	1	85	0	0	0	0	0	0	0	0	0	0	0	0	19	1	0	0	1	5.2	255	1	0	1	2	63.0	0	0
324	1	1	75	0	0	0	0	0	1	1	0	0	0	0	1	10	1	0	0	3	5.7	168	1	0	0	0		0	113
325	1	0	60	1	0	0	0	0	1	1	0	1	1	0	0	28	4	1	0	3	5.9	465	1	0	1	2	150.0	8	8
326	1	1	60	1	0	0	0	0	1	0	0	0	1	0	0	4	1	0	0	1	5.4	237	1	0	1	10	12.2	0	0
327	1	1	34	1	0	0	0	0	1	0	0	1	1	0	1	35	5	1	1	4	6.3	296	1	1	1	2	65.5	0	0
328	1	1	60	0	0	0	1	0	1	0	0	0	0	1	0	9	1	0	0	1	5.7	226	1	0	1	3	16.3	0	0
329	1	1	57	0	0	0	0	0	0	0	0	0	0	0	0	30	1	0	0	1	5.9	222	1	0	1	11	6.1	20	24
330	1	0	68	0	0	0	1	0	0	0	0	0	0	0	0	15	1	0	0	1	5.4	199	1	1	1	9	8.4	0	0
331	1	1	57	0	0	0	0	0	0	0	0	0	1	1	1	38	4	1	0	4	6.2	284	1	0	1	6	18.8	3	15
332	1	1	32	0	0	0	0	0	0	1	0	1	1	0	1	41	5	1	1	2	9	465	1	0	1	15	20.1	237	270.5
333	1	1	45	1	0	0	0	0	1	1	1	1	1	0	0	23	5	1	1	3	5.4	234	1	0	1	4	13.8	2	16
334	1	1	26	0	0	0	0	0	0	0	0	0	0	1	0	27	4	1	0	4	6.9	239	1	0	1	36	1.9	14	14
335	1	1	67	0	0	0	0	0	0	0	0	1	1	1	0	.	1	0	0	1	5.5	103	0	0	0	0		0	0
336	1	1	48	0	0	0	0	0	1	0	0	1	1	0	0	30	5	1	1	1	5.7	242	1	0	1	8	8.8	12	77
337	1	1	56	0	0	0	0	0	0	0	0	0	0	0	0	11	1	0	0	1	5.2	135	0	0	0	0		0	0
338	1	1	52	1	0	0	0	0	0	1	0	0	0	1	0	.	5	1	1	2	9.8	496	1	0	1	16	20.6	38	52
339	1	1	54	0	0	0	1	0	0	0	0	0	0	0	0	16	1	0	0	1	5.8	262	1	0	1	3	35.3	0	48
340	1	1	59	0	0	1	0	0	1	1	1	1	1	0	0	18	5	1	1	3	5.3	201	1	0	1	3	8.0	3	3
341	1	1	76	0	0	0	0	0	0	0	0	1	1	0	0	32	5	1	1	4	7	317	1	0	1	48	.	22	22
342	1	1	16	0	0	1	0	0	0	1	0	0	0	0	0	13	1	0	0	2	9	300	1	0	1	8	20.6	25	46
343	1	1	53	0	0	0	0	0	1	1	1	1	1	0	0	17	5	1	1	2	6.4	233	1	0	1	8	7.6	11	44.5
344	1	1	72	1	0	0	0	0	1	1	0	1	1	1	0	42	5	1	1	2	6.9	377	1	0	1	9	23.1	38	80
345	1	1	76	0	1	0	0	0	0	0	0	0	0	0	0	14	1	0	0	1	5.2	191	1	1	1	21	3.8	0	0
346	1	1	56	0	0	0	0	0	0	0	0	0	0	0	0	78	5	1	1	1	5.5	256	1	0	1	4	20.0	4	6
347	1	0	88	1	0	0	0	0	1	1	1	1	1	1	1	10	2	0	0	2	7.2	320	1	0	1	44	4.1	20	20
348	1	1	51	1	0	0	0	0	0	1	0	1	1	0	1	35	3	1	0	2	11.4	913	1	0	1	15	50.2	246	276
349	1	1	44	0	0	0	0	0	1	0	0	0	0	1	0	>11	1	0	0	1	5.2	340	1	0	1	4	56.8	0	16
350	1	1	52	1	0	0	0	0	1	1	0	1	1	0	0	32	5	1	1	2	7.6	359	1	0	1	14	13.6	33	49
351	1	1	26	0	0	0	0	0	0	0	1	0	0	0	0	25	1	0	0	1	5.4	268	1	0	1	2	54.5	0	0
352	1	1	42	0	0	0	0	0	1	0	0	1	1	1	0	21	1	0	0	1	5.7	331	1	0	1	4	38.3	8	14
353	1	1	57	0	0	0	0	0	1	0	0	0	0	1	1	>19	4	1	0	4	6.3	382	1	0	1	4	61.5	0	0
354	1	0	64	0	0	0	0	0	0	0	0	1	1	1	0	>43	5	1	1	1	5.1	335	1	0	1	6	35.5	5	5
355	1	1	75	0	0	0	0	0	0	0	0	1	1	0	1	65	5	1	1	1	5.7	242	1	0	1	12	7.8	4	4
356	1	1	49	0	0	0	0	0	1	0	0	0	1	1	1	65	1	0	0	4	6.6	400	1	1	1	19	11.6	50	104
357	1	0	65	0	0	0	0	0	0	0	0	1	1	1	0	>24	5	1	1	1	5.9	174	1	0	0	0		0	30
358	1	0	63	1	0	0	1	0	1	1	0	0	0	0	0	12	3	1	0	2	6.6	206	1	1	1	5	12.4	50	52
359	1	0	81	0	0	0	0	0	0	0	0	1	1	1	0	65	5	1	1	1	5.1	223	1	1	1	4	12.3	6	71
360	1	0	22	1	0	0	0	0	1	0	1	0	0	0	1	12	2	0	0	1	4.6	166	0	1	0	0		0	0
361	1	1	19	0	0	0	0	0	0	0	0	1	0	0	0	34	4	1	0	1	5.7	252	1	0	1	15	4.9	32	48
362	1	0	65	1	0	0	0	0	1	1	0	1	1	0	1	35	5	1	1	2	7.3	326	1	0	1	9	16.6	12	53
363	1	1	48	0	0	0	0	0	0	0	0	0	0	1	0	>9	1	0	0	1	5.5	245	1	0	1	3	37.7	0	7
364	1	1	61	0	0	0	0	0	0	0	0	0	0	1	0	80	5	1	1	1	5	373	1	0	1	16	14.0	0	0
365	1	0	41	1	0	0	0	0	0	0	0	1	1	0	0	49	3	1	0	1	5.8	88	0	1	0	0		0	0
366	1	1	52	0	0	0	0	0	0	0	0	0	0	0	0	17	1	0	0	1	5.5	208	1	0	1	2	18.5	0	19
367	1	1	64	0	0	0	0	0	0	0	0	0	1	0	0	76	5	1	1	1	5.5	295	1	1	1	7	16.9	18	20
368	1	1	63	0	0	0	0	0	0	0	0	1	1	1	0	90	5	1	1	1	5.5	283	1	0	1	6	22.8	5	5
369	1	1	56	0	0	0	0	0	1	1	0	0	0	1	1	20	1	0	0	2	6.4	253	1	0	1	6	12.8	3	26
370	1	0	74	0	0	0	0	0	1	0	0	1	1	0	1	42	5	1	1	4	6.3	227	1	0	1	1	51.0	0	14
371	1	1	55	0	0	0	0	0	0	0	0	1	1	0	0	6	4	1	0	1	5.4	232	1	0	1	3	20.3	0	0
372	1	1	81	0	0	0	0	0	0	0	1	0	0	1	0	>20	1	0	0	4	6.5	335	1	0	1	4	40.0	4	73
373	1	1	38	1	0	0	1	0	1	1	1	0	1	0	0	52	5	1	1	2	8.9	459	1	0	1	.	.	0	91
374	1	1	49	0	0	0	0	0	0	0	0	1	1	1	0	53	5	1	1	1	5.3	163	1	1	0	0		0	92.5
375	1	1	41	1	0	0	0	0	0	1	0	1	1	1	1	43	5	1	1	2	7.4	617	1	1	1	20	22.1	124	142.5
376	1	1	34	0	0	0	0	0	0	0	0	0	0	0	0	133	5	1	1	1	5.7	263	1	0	1	.	.	0	25
377	1	0	71	0	0	0	0	0	0	0	0	1	0	1	1	50	5	1	1	1	5.1	266	1	1	1	9	14.7	0	0
378	1	0	36	0	0	0	0	0	0	0	0	0	0	0	0	23	5	1	1	1	5.8	355	1	0	1	6	34.5	0	0
379	1	0	33	0	0	0	0	0	0	0	0	1	1	1	0	2	1	0	0	1	5.3	152	1	0	0	0		0	0
380	1	0	75	1	0	0	1	0	1	0	1	1	1	0	0	13	4	1	0	1	5.1	282	1	0	1	3	39.0	0	0
381	1	0	55	0	0	0	0	0	0	0	0	1	1	0	1	44	5	1	1	1	5.1	533	1	0	1	.	.	0	75
382	1	1	62	0	0	0	0	0	0	0	0	0	0	0	0	50	4	1	0	1	5.1	337	1	0	1	5	45.0	0	0
383	1	1	25	1	0	0	0	0	1	0	0	1	1	1	0	104	5	1	1	4	6.7	336	1	0	1	.	.	0	0
384	1	1	20	0	0	0	0	0	0	0	0	0	0	0	0	15	1	0	0	1	5.9	304	1	0	1	5	29.6	0	16
385	1	1	40	0	0	0	0	0	1	0	0	0	0	0	0	8	1	0	0	1	5.9	279	1	0	1	4	28.8	0	17
386	1	1	52	0	0	0	0	0	0	0	0	1	1	1	0	30	1	0	0	1	5.5	133	0	0	0	0		0	0
387	1	1	29	0	0	0	0	0	0	1	0	1	1	1	1	160	5	1	1	2	9.4	1126	1	0	1	48	.	468	468
388	1	1	46	0	0	0	0	0	0	0	0	1	1	1	1	>33	4	1	0	1	4.8	229	1	0	1	15	4.1	0	0
389	1	1	81	0	0	0	0	0	0	0	0	0	0	1	1	>55	5	1	1	1	5.8	304	1	0	1	29	5.4	0	16
390	1	1	40	0	0	0	0	0	0	0	0	1	1	0	0	44	5	1	1	1	5.3	300	1	1	1	5	32.8	0	0
391	1	1	53	0	0	0	0	0	0	0	0	1	1	0	1	55	5	1	1	1	5.4	211	1	0	1	21	3.0	2	2
392	1	1	61	1	0	0	1	0	1	0	0	1	1	0	0	30	5	1	1	1	5.4	305	1	0	1	18	7.2	0	0
393	1	1	63	0	0	0	0	0	0	0	0	1	0	0	0	22	5	1	1	1	5.5	305	1	1	1	6	25.0	0	0
394	1	1	70	0	0	0	0	0	0	1	1	1	1	1	0	>47	5	1	1	2	6.4	551	1	0	1	34	11.3	170	193
395	1	1	71	0	0	0	0	0	0	0	0	1	1	0	1	28	4	1	0	1	5.7	247	1	0	1	8	11.6	0	0

396	1	1	55	0	0	0	0	0	1	1	1	0	1	0	0	36	5	1	1	3	5.4	274	1	0	1	12	10.1	28	44
397	1	0	54	1	0	0	0	0	0	0	1	1	1	0	1	46	5	1	1	1	5.1	169	1	0	0	0		0	4
398	1	0	67	1	0	0	0	0	1	0	0	1	1	1	0	109	5	1	1	1	5.7	277	1	0	1	18	8.9	6	6
399	1	1	54	0	0	0	0	0	0	0	0	0	0	0	0	23	1	0	0	4	6.1	201	1	0	1	25	3.0	0	0
400	1	1	58	0	0	0	0	0	0	0	0	0	0	0	0	67	1	0	0	1	5.3	290	1	0	1	19	6.2	0	0
401	1	1	36	1	0	0	0	0	0	0	0	0	0	1	0	>13	1	0	0	4	6	232	1	0	1	6	19.5	0	0
402	1	1	63	0	0	1	0	0	1	1	0	0	1	0	1	88	5	1	1	2	6.1	385	1	1	1	16	13.3	31	47.5
403	1	0	76	0	0	0	0	0	0	1	1	1	0	0	0	54	1	0	0	3	5.3	166	1	1	0	0		0	38
404	1	1	71	0	0	0	0	0	0	0	0	1	0	0	0	28	4	1	0	4	6.3	281	1	0	1	4	48.5	2	2
405	1	1		0	1	0	0	0	1	0	0	0	0	1	0	43	4	1	0	4	6.1	258	1	1	1	8	9.9	0	0
406	1	1		1	0	0	1	0	1	0	0	1	1	0	1	36	5	1	1	1	5.8	265	1	0	1	6	19.8	0	0
407	1	1		1	1	1	0	0	1	0	0	1	0	0	0	20	1	0	0	4	6	272	1	0	1	7	14.0	0	0
