# Supplementary material for: Glucose control and outcomes in diabetic and nondiabetic patients treated with targeted temperature management after cardiac arrest
Source: PLoS One. 2024 Feb 8;19(2):e0298632. doi: 10.1371/journal.pone.0298632 (PMC10852315; doi:10.1371/journal.pone.0298632)
Supplement: S2 File — (PDF) [file pone.0298632.s002.pdf]

S2. Glucose levels of patients by category used for Fig 2

		0h	1h	2h	3h	4h	5h	6h	7h	8h	9h	10h	11h	12h	13h	14h	15h	16h	17h	18h	19h	20h	21h	22h	23h	24h
0: No diabetes	mean	262.51	244.10	234.67	221.56	204.14	194.40	181.58	171.69	162.48	161.49	154.71	156.19	156.46	157.50	151.22	150.97	145.32	148.48	147.58	148.64	145.07	145.39	142.12	141.65	141.51
	lower CI	249.10	211.80	216.52	206.95	192.04	183.32	172.38	162.14	154.07	153.42	147.63	148.06	148.51	149.13	143.52	142.16	137.94	139.26	139.76	141.21	138.08	136.32	132.65	133.71	134.69
	higher CI	275.91	276.40	252.82	236.18	216.24	205.48	190.78	181.25	170.89	169.56	161.80	164.31	164.42	165.88	158.92	159.78	152.70	157.70	155.40	156.07	152.07	154.46	151.59	149.59	148.33
	SD	6.799				6.120				4.258				4.026				3.733				3.536				3.448
1: Inadequatly controlled diabetes	mean	357.37	313.37	299.15	312.29	287.08	264.51	241.04	219.79	222.85	209.90	207.88	202.54	194.09	201.79	187.43	184.06	176.42	188.50	173.32	179.27	179.94	182.00	172.54	179.84	181.69
	lower CI	313.67	269.90	264.86	265.86	260.71	233.99	219.08	196.74	197.33	184.79	181.76	180.16	174.92	176.00	164.82	156.65	154.82	159.31	150.24	150.62	154.88	153.66	145.92	146.53	154.13
	higher CI	401.06	356.83	333.45	358.72	313.45	295.03	263.00	242.83	248.38	235.01	234.00	224.91	213.26	227.58	210.05	211.47	198.01	217.69	196.40	207.93	205.00	210.34	199.17	213.16	209.26
	SD	21.857				13.122				12.730				9.557				10.762				12.473				13.710
2: Controlled diabetes	mean	267.20	276.25	213.88	240.42	213.40	194.18	225.22	206.57	189.88	167.00	179.40	150.53	142.61	135.93	130.29	142.19	151.50	147.38	140.71	147.27	147.46	145.08	150.55	177.43	175.00
	lower CI	212.32	172.67	137.03	209.26	160.32	147.86	161.10	139.05	145.05	129.51	140.67	126.14	124.08	112.46	109.55	119.89	125.28	117.98	107.69	122.08	114.82	122.33	103.90	75.48	87.46
	higher CI	322.08	379.83	290.72	271.58	266.48	240.50	289.34	274.09	234.70	204.49	218.13	174.92	161.14	159.41	151.02	164.48	177.72	176.77	173.74	172.46	180.11	167.82	197.19	279.38	262.54
	SD	26.223				23.463				21.029				8.784				12.300				14.983				40.523
3: Unrecognized diabetes	mean	270.69	243.25	265.42	280.45	238.45	231.85	211.26	218.67	198.62	179.61	178.16	169.37	178.63	177.22	174.05	172.40	171.33	159.23	159.50	157.52	157.64	161.00	163.89	159.89	158.97
	lower CI	243.79	128.75	215.09	240.52	200.35	197.61	180.25	191.55	175.06	159.56	148.29	144.76	155.55	155.07	151.32	149.64	150.12	135.21	140.59	137.72	137.49	131.66	139.99	133.28	141.34
	higher CI	297.58	357.75	315.75	320.38	276.56	266.10	242.26	245.78	222.17	199.65	208.04	193.97	201.70	199.36	196.79	195.16	192.54	183.24	178.41	177.31	177.79	190.34	187.80	186.51	176.59
	SD	13.388				18.658				11.637				11.410				10.477				9.893				8.641

		0h	1h	2h	3h	4h	5h	6h	7h	8h	9h	10h	11h	12h	13h	14h	15h	16h	17h	18h	19h	20h	21h	22h	23h	24h
0: No diabetes	0.25	186				158.25				130.75				126.5				118				120				117.75
	median	258	228.5	222.5	195.5	207	182	168	161	155.5	153.5	148	146	154	147	141	138	136.5	138	138	138	136.5	135	136	134	131
	0.75	311.75				249.25				193.25				182				166				159.5				152
1: Inadequatly controlled diabetes	0.25	250				224.5				155.5				152.75				128.25				134.25				131.25
	median	312.5	331	283	250	283.5	263	236	205	194.5	189.5	187	190.5	199.5	187.5	171	158	157	159	156	150.5	155	154	149	147	144.5
	0.75	366.75				342				270.25				239.25				196				190.75				176.5
2: Controlled diabetes	0.25	157.5				156.5				115.75				102.25				100				99.25				84.75
	median	207	229	233	261	223	186	197	175	145.5	175	164	143	117.5	140	129	140	119.5	138.5	136	140	162	135	146	131.5	136
	0.75	291				318.75				221				199.5				244				204.5				205.25
3: Unrecognized diabetes	0.25	192.75				142.75				189.25				130.75				134.75				132.25				123.5
	median	257	149	181	163	168.5	226	205	198	231.5	176.5	153	156	157.5	160	169.5	159	156.5	148	145.5	148	140	135	147.5	144.5	149
	0.75	355.25				264				276				212				187				177.75				196.5
