# Supplementary material for: Glucose control and outcomes in diabetic and nondiabetic patients treated with targeted temperature management after cardiac arrest
Source: PLoS One. 2024 Feb 8;19(2):e0298632. doi: 10.1371/journal.pone.0298632 (PMC10852315; doi:10.1371/journal.pone.0298632)
Supplement: S1 Table — (DOCX) [file pone.0298632.s003.docx]

| Poor neurologic outcome | OR | 95% CI | *p value* | No survival | OR | 95% CI | *p value* |
| --- | --- | --- | --- | --- | --- | --- | --- |
| Male | 1.359 | 0.788–2.346 | 0.270 |  | 0.848 | 0.516–1.393 | 0.514 |
| Age ≥65 | 2.725 | 1.563–4.749 | <0.001 |  | 2.774 | 1.686–4.565 | <0.001 |
| History of previous cardiac arrest | 0.642 | 0.106–3.902 | 0.630 |  | 0.167 | 0.018–1.512 | 0.111 |
| History of AMI | 1.016 | 0.405–2.552 | 0.973 |  | 0.883 | 0.376–2.077 | 0.776 |
| History of angina pectoris | 0.600 | 0.248–1.453 | 0.258 |  | 0.667 | 0.280–1.585 | 0.359 |
| History of CHF | 2.628 | 0.312–22.120 | 0.374 |  | 1.732 | 0.331–9.059 | 0.515 |
| History of hypertension | 1.543 | 0.925–2.572 | 0.096 |  | 1.638 | 1.022–2.625 | 0.040 |
| History of DM | 2.892 | 1.515–5.521 | 0.001 |  | 2.493 | 1.433–4.339 | 0.001 |
| History of renal disease | 1.808 | 0.715–4.569 | 0.211 |  | 2.417 | 1.006–5.806 | 0.048 |
| Noncardiac cause arrest | 9.786 | 4.969–19.271 | <0.001 |  | 4.832 | 2.924–7.983 | <0.001 |
| Initial nonshockable rhythm | 9.852 | 5.724–16.957 | <0.001 |  | 6.889 | 4.205–11.284 | <0.001 |
| Unwitnessed arrest | 2.299 | 1.327–3.982 | 0.003 |  | 2.199 | 1.344–3.598 | 0.002 |
| No bystander CPR | 1.533 | 0.928–2.533 | 0.095 |  | 1.601 | 1.007–2.546 | 0.047 |
| Total anoxic time | 1.062 | 1.042–1.083 | <0.001 |  | 1.056 | 1.039–1.074 | <0.001 |
| Initial HbA1c | 1.229 | 0.971–1.556 | 0.086 |  | 1.266 | 1.021–1.571 | 0.032 |
| Admission glucose | 1.004 | 1.002–1.007 | 0.001 |  | 1.003 | 1.000–1.005 | 0.016 |
| Mean glucose during 48 h | 1.010 | 1.004–1.017 | 0.002 |  | 1.010 | 1.004–1.016 | 0.001 |
| Median glucose during 48 h | 1.008 | 1.002–1.014 | 0.007 |  | 1.008 | 1.003–1.014 | 0.002 |
| Range glucose during 48 h | 1.005 | 1.002–1.008 | <0.001 |  | 1.003 | 1.001–1.005 | 0.003 |
| At least one hyperglycemic episode during 48 h | 1.512 | 0.567–4.022 | 0.407 |  | 1.178 | 0.453–3.066 | 0.737 |
| At least one hypoglycemic episode during 48 h | 3.067 | 1.450–6.491 | 0.003 |  | 2.577 | 1.376–4.827 | 0.003 |
| Hyperglycemia at admission | 1.277 | 0.692–2.358 | 0.434 |  | 0.968 | 0.536–1.746 | 0.913 |
| Time to target glucose | 1.012 | 0.981–1.043 | 0.469 |  | 1.008 | 0.980–1.037 | 0.575 |
| Glucose reducing rate | 1.007 | 0.997–1.017 | 0.160 |  | 1.004 | 0.997–1.010 | 0.305 |
| Total insulin dose to target glucose | 1.004 | 0.997–1.011 | 0.323 |  | 1.003 | 0.997–1.009 | 0.358 |
| Cumulative insulin requirement during 48 h | 1.003 | 0.998–1.008 | 0.223 |  | 1.003 | 0.999–1.007 | 0.211 |
| Categorization |  |  |  |  |  |  |  |
| No diabetes | ref | ref | ref |  | ref | ref | ref |
| Inadequately controlled diabetes | 2.364 | 1.157–4.829 | 0.018 |  | 2.121 | 1.137–3.955 | 0.018 |
| Controlled diabetes | 4.500 | 1.014–19.976 | 0.048 |  | 3.135 | 1.012–9.715 | 0.048 |
| Unrecognized diabetes | 0.714 | 0.380–1.343 | 0.296 |  | 0.697 | 0.376–1.292 | 0.251 |

**Supplementary Table 1. Univariate logistic regression analysis**

Abbreviations: AMI, acute myocardial infarction; CHF, congestive heart failure; CPR, cardiopulmonary resuscitation; OR, odds ratio; CI, confidence interval.
