# Supplementary material for: Glucose control and outcomes in diabetic and nondiabetic patients treated with targeted temperature management after cardiac arrest
Source: PLoS One. 2024 Feb 8;19(2):e0298632. doi: 10.1371/journal.pone.0298632 (PMC10852315; doi:10.1371/journal.pone.0298632)
Supplement: Attachment — Submitted filename: Response to reviewers.docx [file pone.0298632.s004.docx]

**Reviewer #1**

**───────────────────────────────────────────────────────────────**

Dear authors, this is an interesting study regarding the impact of glycemic control in patients with pre existing DM and those without in neuroprotection after survivors of cardiac arrest.

1-1) Introduction: could benefit from adding a hypothesis and to provide more scientific rationale for it. The following paragraph "In this study, we classified patients into two groups: those who had been diagnosed with diabetes and those who had not. We then further classified patients in these two groups into subgroups based on their preexisting levels of glucose control using glycated hemoglobin (HbA1c). Our final aim was to compare the glucose hemodynamics of these subgroups and, if differences exist, provide a basis for more ideal glucose control for each group. We used HbA1c to assess preexisting levels of glucose control [27–29]. A cutoff value ofHbA1c 6.0% was set to determine whether glucose levels were well controlled because we believed that different standards might be appropriate for acute-care settings such as emergency rooms." contains many elements that belong in the methods section. In the introduction it would be provides the hypothesis, study rationale and aims.

2-1) Methods: Well written but I would add the information of the last paragraph of the introduction in the methods section.

*We appreciate the reviewer’s suggestion. We edited the introduction and the methods as you commented for the concision and precision of the article.*

- *Our hypothesis was ~~We wondered if~~ there would be ~~were any~~ differences in outcomes and variables according to the glycemic status of patients and whether they were diagnosed with diabetes.*

2-2) Please provide the reference for you cutoff for hypoglycemia.

*We thoroughly agree with the reviewer’s comment on the lack of the citation. We added the reference of the hypoglycemia criteria.*

- *Hypoglycemia was defined as a blood glucose value less than 72 mg/dL [28].*
- *[28] Davis SN, Shavers C, MosquedaGarcia R, Costa F. Effects of differing antecedent hypoglycemia on subsequent counterregulation in normal humans. Diabetes. 1997;46(8):1328-35. doi: DOI 10.2337/diabetes.46.8.1328. PubMed PMID: WOS:A1997XM40000012.*

2-3) And please provide the rational for using HbA1c of 6 % instead of 6.5%.

*We appreciate the reviewer’s suggestion. It seems that the explanation as to why we set 6.0% as the standard was insufficient. According to the reviewer’s opinion for clarity, we tried to explain in more detail why we set 6.0% instead of 6.5%.*

- *Clinical guidelines generally advocate for an HbA1c threshold of ≥6.5% for diagnosing diabetes and a range of 5.7 to 6.4% for identifying prediabetes. However, Silverman et al. have suggested that in acute-care settings such as emergency departments, an HbA1c of 5.7% is the optimal screening cutoff for prediabetes, while 6% is optimal for diagnosing diabetes [33, 34]. Consequently, we established a cutoff value of HbA1c at 6.0% to assess the adequacy of glucose control and diagnose diabetes.*

2-4) Statistical analysis: the multivariate analysis should also include known risk factors for poor outcome in cardiac arrest and not just with a p<0.05 in the univariate analysis as this approach is not recommended. (DOI 10.1513/AnnalsATS.201808-564PS)

*We totally agree with the reviewer’s thoughtful comments about the multivariate analysis. As the reviewer suggested, we included a set of potential confounders derived from published studies. These confounders were witness status, bystander CPR, and the initial presence of a shockable rhythm. We performed multivariate logistic regression with all these variables. The results were edited and the method section was also supplemented.*

- *To assess independent predictors of poor neurologic outcome and death, we included all variables with p-values less than 0.05 in univariate analyses, along with a set of potential confounders derived from published studies, in a multivariate logistic regression analysis. The considered confounders included noncardiac cause arrest, initial nonshockable rhythm, unwitnessed arrest and no bystander CPR [3, 37, 38]. ~~the~~ The odds ratios (ORs) with 95% confidence intervals (CIs) were calculated.	- Method*
- ***Table 4. Multivariate logistic regression analysis***

| *Poor neurologic outcome* | *OR* | *95% CI* | *p value* |
| --- | --- | --- | --- |
| *Age ≥65* | *2.780* | *1.200–6.445* | *0.017* |
| *Noncardiac cause arrest* | *5.239* | *2.113–12.989* | *<0.001* |
| *Initial nonshockable rhythm* | *6.439* | *2.950–14.055* | *<0.001* |
| *Unwitnessed arrest* | *0.874* | *0.357–2.137* | *0.768* |
| *No bystander CPR* | *0.689* | *0.323–1.471* | *0.336* |
| *Total anoxic time* | *1.063* | *1.038–1.087* | *<0.001* |
| *Admission glucose* | *1.004* | *0.999–1.009* | *0.154* |
| *Mean glucose during 48 h* | *0.995* | *0.960–1.032* | *0.793* |
| *Median glucose during 48 h* | *1.014* | *0.983–1.046* | *0.385* |
| *Range glucose during 48 h* | *1.001* | *0.996–1.006* | *0.699* |
| *At least one hypoglycemic episode during 48 h* | *3.522* | *1.096–11.322* | *0.035* |
| *Categorization* |  |  | *0.573* |
| *No diabetes* | *ref* | *ref* | *ref* |
| *Inadequately controlled diabetes* | *1.041* | *0.361–3.005* | *0.940* |
| *Controlled diabetes* | *2.625* | *0.352–19.568* | *0.346* |
| *Unrecognized diabetes* | *0.636* | *0.245–1.650* | *0.352* |
|  |  |  |  |
| *No survival* |  |  |  |
| *Age ≥65* | *2.242* | *1.061–4.739* | *0.034* |
| *History of hypertension* | *2.124* | *1.012–4.461* | *0.046* |
| *History of renal disease* | *1.166* | *0.345–3.941* | *0.805* |
| *Noncardiac cause arrest* | *2.569* | *1.234–5.351* | *0.012* |
| *Initial nonshockable rhythm* | *4.838* | *2.371–9.871* | *<0.001* |
| *Unwitnessed arrest* | *1.149* | *0.542–2.436* | *0.718* |
| *No bystander CPR* | *0.943* | *0.489–1.821* | *0.862* |
| *Total anoxic time* | *1.059* | *1.039–1.080* | *<0.001* |
| *Admission glucose* | *1.000* | *0.996–1.004* | *0.867* |
| *Mean glucose during 48 h* | *1.013* | *0.982–1.045* | *0.426* |
| *Median glucose during 48 h* | *0.999* | *0.973–1.026* | *0.955* |
| *Range glucose during 48 h* | *0.999* | *0.994–1.004* | *0.683* |
| *At least one hypoglycemic episode during 48 h* | *2.509* | *0.959–6.565* | *0.061* |
| *Categorization* |  |  | *0.385* |
| *No diabetes* | *ref* | *ref* | *ref* |
| *Inadequately controlled diabetes* | *0.829* | *0.315–2.179* | *0.703* |
| *Controlled diabetes* | *1.522* | *0.363–6.388* | *0.566* |
| *Unrecognized diabetes* | *0.479* | *0.186–1.229* | *0.126* |

*- Result*

- *~~While t~~The patient categories did not have an impact in multivariate analysis~~, initial glucose level was an independent predictor of 6-month poor neurological outcome, consistent with previous studies [20]~~.	- Discussion*

3-1) Results: The following sentence "HbA1c, admission glucose, mean and median values of initial glucose level were significantly higher in the groups of inadequately controlled diabetes and unrecognized diabetes."seems redundant since HbA1c was used to classify the different subgroups.

*We thoroughly agree with the reviewer’s point of view.* *We edited the sentences for brevity as the reviewer suggested and corrected* *the incorrectly mentioned word.*

- *~~HbA1c,~~ Admission glucose, mean and median values of ~~initial~~ glucose level were significantly higher in the groups of inadequately controlled diabetes and unrecognized diabetes.*

3-2) It would also be interesting to know if the inulin doses were different in the groups regarding the use of oral DM medication.

*We sincerely agree with you and thank you for your insightful comment. At first, we also wanted to analyze the oral medication or insulin that patients diagnosed with diabetes had previously received, but there was a lot of missing information, so we were unable to analyze the information in this study. Reflecting the thoughtful point, the following information was added to the discussion section.*

- *Forth, a substantial portion of data regarding oral medications or insulin usage before cardiac arrest was unavailable for patients diagnosed with diabetes, leading to an inadequate basis for analysis.*

3-3) In the multivariable analysis I don't think the authors should add both the variable different subgroups and the variable HbA1c, since HbA1C was use to categorize the different subgroups

*We deeply appreciate the reviewer’s thoughtful comments. As suggested by the authors, HbA1C was excluded from the multivariate analysis as it was used to classify different subgroups. We re-conducted the statistical analysis, including known risk factors following the advice provided in points above (2-4). The results have been reflected in the Results section.*

4) Discussion: well written. I have some comments: While I agree that stress induced hyperglycemia is a great component to justify hyperglycemia in post cardiac arrest and this should merit a paragraph I the discussion, this is true for all subgroups and I am not sure that it can be used to explain the differences in the difficult to achieve glycemic control the different groups. Another interesting point is that although all patient underwent TTM no data is given regarding body temperature and other TTM data as this may also have influenced glycemic metabolic. this is acknowledged by the authors in the limitations.

*We fully agree with you and thank you for the insightful comments. Our ultimate goal of categorization was to help achieve a better outcome by providing tailored glucose control based on patients’ glycemic status rather than a uniform treatment. We believe that further studies, including prospective studies or RCTs, will help better glucose control and improve outcomes if they secure the legitimacy of personalized treatment.*

**Reviewer #2**

**───────────────────────────────────────────────────────────────**

I have some critiques authors need to address.

1) Objective is clear that authors categorized patients with glycemic condition and diabetes diagnosis. Then, authors evaluated association between outcomes and categorization as well as glycemic conditions. Conclusion is incomplete. Especially in the abstract section, it is vague what authors want to convey to the readers. Categorization was a primary objective. However, it did not predict outcomes. Authors should touch on the conclusions. Authors also should suggest or indicate treatment plan based on the discoveries of glycemic hemodynamics.

*We do appreciate the reviewer's suggestion on the vague conclusion. As per the reviewer's opinion, we strengthened the contents of the treatment on hyperglycemia in the discussion section and tried to mention the contents more clearly in the abstract and conclusion section.*

- *Surviving patients following cardiac arrest exhibited variations in glucose hemodynamics and outcomes according to the categories based on their preexisting diabetes status and glycemic condition. Specifically, even experiencing a single episode of hypoglycemia during the acute phase could have an influence on unfavorable neurological outcomes. While the classification did not directly affect neurological outcomes, the present results indicate the need for a customized approach to glucose control based on these categories.	- Abstract*
- *Therefore, stabilizing glucose variability according to a patient's condition may affect the outcomes in addition to their initial glucose level. Our study suggests that different approaches may be necessary based on the patient's glycemic status and presence of diabetes. Despite numerous investigations into conventional versus individualized glucose control for critically ill patients, conclusive evidence remains elusive. [26, 45]. A specific study on diabetic ketoacidosis patients indicated that a combination of intravenous insulin and subcutaneous glargine showed a trend towards faster resolution and shorter hospital stays, though statistically insignificant [46]. This highlights the necessity for more research on insulin types, doses, and target glucose levels tailored to individual characteristics, with a crucial emphasis on preventing hypoglycemia.	- Discussion*
- *Patients who survived after cardiac arrest showed differences in glucose hemodynamics and outcomes depending on their preexisting diabetes status and glycemic status. In particular, even a single occurrence of hypoglycemia during the acute period could have an impact on poor neurological outcomes. Although this categorization did not directly influence the neurological outcomes, we could suggest future studies are warranted to implement a tailored glucose control approach in each group.*

*- Conclusion*

2) Regarding authors discussion about worse outcomes in controlled diabetes patients and best outcomes in unrecognized diabetes patients, are the potential reasons inadequate power due to small sample size and age bias?

*We fully agree with the thoughtful comments of the reviewer. Despite various factors influencing the neurological outcome, as the reviewer rightly pointed out, the small sample size and the increasing comorbidity with patient age might have had an impact on the outcome. We cautiously predicted that these factors could have influenced the outcome. We have incorporated the reviewer’s valuable input into the discussion section.*

- *In the comparison of outcomes among subgroups, both neurological outcome and mortality were highest in controlled diabetes patients and lowest in unrecognized diabetes patients, and the reason for this is unclear. The potential reasons for poor outcomes in controlled diabetes patients and good outcomes in unrecognized diabetes patients may be attributed to inadequate statistical power due to a small sample size and potential age bias in the study population.*

3-1) How did Author define range glucose? It is not described in method section.

*As pointed out by the reviewer, the definition has been added to the method for better understanding. As per the reviewer's comment, we provided the definition of the range glucose in the revised manuscript.*

- *The glucose variability included the median, range, and mean value of the glucose. The range was calculated as the difference between the maximum and minimum blood glucose values during 48 h [20].*

3-2) Authors mention range glucose in discussion section with univariate analysis data only. Is there any reason why authors do not conduct multivariate analysis for this marker?

*We deeply appreciate the reviewer’s thoughtful comments. In the univariate analysis considering individual factors, admission glucose, mean, median, and the range of glucose during the initial 48 hours remained statistically significant. However, when attempting a more comprehensive multivariate analysis, these variables lost statistical power, and thus, their results were not originally included in the manuscript before the revision. In response to the suggestions from Reviewer 1 and Reviewer 2 to incorporate established risk factors for adverse outcomes in cardiac arrest and variables with a p-value less than 0.05 in the univariate analysis, we conducted a multivariate analysis with these specified variables. The conclusive outcomes are presented in Table 4. To offer a thorough overview, we also included supplementary Table 1, delineating the findings from the univariate logistic regression analysis.*

- ***Table 4. Multivariate logistic regression analysis***

| *Poor neurologic outcome* | *OR* | *95% CI* | *p value* |
| --- | --- | --- | --- |
| *Age ≥65* | *2.780* | *1.200–6.445* | *0.017* |
| *Noncardiac cause arrest* | *5.239* | *2.113–12.989* | *<0.001* |
| *Initial nonshockable rhythm* | *6.439* | *2.950–14.055* | *<0.001* |
| *Unwitnessed arrest* | *0.874* | *0.357–2.137* | *0.768* |
| *No bystander CPR* | *0.689* | *0.323–1.471* | *0.336* |
| *Total anoxic time* | *1.063* | *1.038–1.087* | *<0.001* |
| *Admission glucose* | *1.004* | *0.999–1.009* | *0.154* |
| *Mean glucose during 48 h* | *0.995* | *0.960–1.032* | *0.793* |
| *Median glucose during 48 h* | *1.014* | *0.983–1.046* | *0.385* |
| *Range glucose during 48 h* | *1.001* | *0.996–1.006* | *0.699* |
| *At least one hypoglycemic episode during 48 h* | *3.522* | *1.096–11.322* | *0.035* |
| *Categorization* |  |  | *0.573* |
| *No diabetes* | *ref* | *ref* | *ref* |
| *Inadequately controlled diabetes* | *1.041* | *0.361–3.005* | *0.940* |
| *Controlled diabetes* | *2.625* | *0.352–19.568* | *0.346* |
| *Unrecognized diabetes* | *0.636* | *0.245–1.650* | *0.352* |
|  |  |  |  |
| *No survival* |  |  |  |
| *Age ≥65* | *2.242* | *1.061–4.739* | *0.034* |
| *History of hypertension* | *2.124* | *1.012–4.461* | *0.046* |
| *History of renal disease* | *1.166* | *0.345–3.941* | *0.805* |
| *Noncardiac cause arrest* | *2.569* | *1.234–5.351* | *0.012* |
| *Initial nonshockable rhythm* | *4.838* | *2.371–9.871* | *<0.001* |
| *Unwitnessed arrest* | *1.149* | *0.542–2.436* | *0.718* |
| *No bystander CPR* | *0.943* | *0.489–1.821* | *0.862* |
| *Total anoxic time* | *1.059* | *1.039–1.080* | *<0.001* |
| *Admission glucose* | *1.000* | *0.996–1.004* | *0.867* |
| *Mean glucose during 48 h* | *1.013* | *0.982–1.045* | *0.426* |
| *Median glucose during 48 h* | *0.999* | *0.973–1.026* | *0.955* |
| *Range glucose during 48 h* | *0.999* | *0.994–1.004* | *0.683* |
| *At least one hypoglycemic episode during 48 h* | *2.509* | *0.959–6.565* | *0.061* |
| *Categorization* |  |  | *0.385* |
| *No diabetes* | *ref* | *ref* | *ref* |
| *Inadequately controlled diabetes* | *0.829* | *0.315–2.179* | *0.703* |
| *Controlled diabetes* | *1.522* | *0.363–6.388* | *0.566* |
| *Unrecognized diabetes* | *0.479* | *0.186–1.229* | *0.126* |

*- Result*

4) CPC is not defined.

*We appreciate the reviewer’s comment on the lack of CPC definition. According to the reviewer's comment, we defined CPC in the method section.*

- *The primary outcomes were neurological outcome defined by a Cerebral Performance Category (CPC) and death at 6 months after CA. The CPC scale spans from 1 to 5: 1 signifies good cerebral performance or slight disability, 2 indicates moderate disability with independence in daily activities, 3 denotes severe disability requiring assistance from others, 4 signifies a coma or vegetative state, and 5 signifies death or brain death. A good neurological outcome was defined as CPC 1–2, while a poor neurological outcome was defined as a CPC score of 3–5.*

5) Method section: “initial HbA1c of 6.0% or less” does this mean initial HbA1c less than 6.0%?

*We deeply appreciate the reviewer’s thoughtful comments. Generally, our center set the target temperature as 33℃. Ninety percent of the patients received TTM at 33°C, while the remaining received TTM at 36°C. As the reviewer suggested, we have added the target temperature column to the table included in the results. Additionally, we have mentioned the details in the revised manuscript.*

- *The controlled diabetes was defined as patients with diabetes before CA with an initial HbA1c ~~of 6.0%~~ ~~or~~ less than 6.0%. The unrecognized diabetes patients were never diagnosed with diabetes but had an initial HbA1c of 6.0% or higher. Finally, no diabetes was defined as patients were never diagnosed with diabetes but had an initial HbA1c ~~of 6.0% or~~ less than 6.0%.*

6) Figure 2 does not have foot note. Are the data median +/- IQR?

*We appreciate the reviewer’s comment on the lack of the footnote. We tried to show the glucose trend of each group, the data of Fig 2 are mean value with standard deviation. We added the footnote as figure legends in revised manuscript.*

- ***Fig 2. Time to reach target glucose according to the categories****. Each trend line represents the declining trend in glucose levels for each group, with mean values and standard deviations indicated.*
